# Supplementary material for: Modeling glioblastoma heterogeneity as a dynamic network of cell states
Source: Mol Syst Biol. 2021 Sep 16;17(9):e10105. doi: 10.15252/msb.202010105 (PMC8444284; doi:10.15252/msb.202010105)
Supplement: Supplementary file 6 — Source Data for Figure 5 [file MSB-17-e10105-s004.zip › Figure5A_sourcedata/GSEA_3017/nosigdb_stateA.GseaPreranked.1622795026485/RICHARDS_NOWAKOWSKI_SCIENCE_2017_MGE_DIV_UPREG.html]

Details for gene set RICHARDS\_NOWAKOWSKI\_SCIENCE\_2017\_MGE\_DIV\_UPREG[GSEA]

|  || Dataset | state53017 |
| Phenotype | NoPhenotypeAvailable |
| Upregulated in class | na\_pos |
| GeneSet | RICHARDS\_NOWAKOWSKI\_SCIENCE\_2017\_MGE\_DIV\_UPREG |
| Enrichment Score (ES) | 0.4165934 |
| Normalized Enrichment Score (NES) | 3.4025822 |
| Nominal p-value | 0.0 |
| FDR q-value | 0.0 |
| FWER p-Value | 0.0 |
Table: GSEA Results Summary

  

Fig 1: Enrichment plot: RICHARDS\_NOWAKOWSKI\_SCIENCE\_2017\_MGE\_DIV\_UPREG      
 Profile of the Running ES Score & Positions of GeneSet Members on the Rank Ordered List

  

| PROBE | GENE SYMBOL | GENE\_TITLE | RANK IN GENE LIST | RANK METRIC SCORE | RUNNING ES | CORE ENRICHMENT || 1 | CDK1 |  |  | 3 | 1.006 | 0.0124 | Yes |
| 2 | MKI67 |  |  | 4 | 0.926 | 0.0272 | Yes |
| 3 | KIF11 |  |  | 6 | 0.870 | 0.0399 | Yes |
| 4 | UBE2C |  |  | 7 | 0.793 | 0.0525 | Yes |
| 5 | MCM10 |  |  | 9 | 0.752 | 0.0633 | Yes |
| 6 | KIF20B |  |  | 10 | 0.749 | 0.0752 | Yes |
| 7 | BUB3 |  |  | 12 | 0.740 | 0.0858 | Yes |
| 8 | DBI |  |  | 17 | 0.683 | 0.0919 | Yes |
| 9 | HIST1H4C |  |  | 21 | 0.659 | 0.0988 | Yes |
| 10 | ASPM |  |  | 27 | 0.639 | 0.1029 | Yes |
| 11 | DNAJC9 |  |  | 34 | 0.615 | 0.1055 | Yes |
| 12 | CCNB1 |  |  | 37 | 0.599 | 0.1126 | Yes |
| 13 | CKS2 |  |  | 40 | 0.587 | 0.1196 | Yes |
| 14 | NCAPG |  |  | 44 | 0.556 | 0.1248 | Yes |
| 15 | HJURP |  |  | 47 | 0.539 | 0.1310 | Yes |
| 16 | CKS1B |  |  | 50 | 0.530 | 0.1370 | Yes |
| 17 | DBF4 |  |  | 55 | 0.514 | 0.1404 | Yes |
| 18 | SMC3 |  |  | 58 | 0.504 | 0.1460 | Yes |
| 19 | KIF14 |  |  | 59 | 0.503 | 0.1540 | Yes |
| 20 | MASTL |  |  | 60 | 0.499 | 0.1620 | Yes |
| 21 | CENPE |  |  | 62 | 0.496 | 0.1687 | Yes |
| 22 | MIS18BP1 |  |  | 64 | 0.495 | 0.1754 | Yes |
| 23 | TPX2 |  |  | 66 | 0.488 | 0.1819 | Yes |
| 24 | MYBL2 |  |  | 69 | 0.486 | 0.1873 | Yes |
| 25 | HNRNPF |  |  | 70 | 0.485 | 0.1950 | Yes |
| 26 | HIST1H1B |  |  | 77 | 0.475 | 0.1953 | Yes |
| 27 | VRK1 |  |  | 82 | 0.470 | 0.1980 | Yes |
| 28 | UBE2T |  |  | 83 | 0.470 | 0.2055 | Yes |
| 29 | DUT |  |  | 87 | 0.466 | 0.2093 | Yes |
| 30 | TUBB4B |  |  | 93 | 0.462 | 0.2106 | Yes |
| 31 | CENPF |  |  | 94 | 0.461 | 0.2180 | Yes |
| 32 | TOP2A |  |  | 95 | 0.457 | 0.2253 | Yes |
| 33 | HIST1H1D |  |  | 98 | 0.456 | 0.2301 | Yes |
| 34 | PRTFDC1 |  |  | 104 | 0.448 | 0.2312 | Yes |
| 35 | ZWINT |  |  | 106 | 0.447 | 0.2371 | Yes |
| 36 | AURKB |  |  | 108 | 0.446 | 0.2430 | Yes |
| 37 | SEPHS1 |  |  | 114 | 0.437 | 0.2440 | Yes |
| 38 | CDCA8 |  |  | 115 | 0.437 | 0.2509 | Yes |
| 39 | KIF23 |  |  | 116 | 0.436 | 0.2579 | Yes |
| 40 | MELK |  |  | 123 | 0.427 | 0.2574 | Yes |
| 41 | BUB1B |  |  | 129 | 0.421 | 0.2581 | Yes |
| 42 | HELLS |  |  | 135 | 0.417 | 0.2587 | Yes |
| 43 | GTPBP4 |  |  | 137 | 0.414 | 0.2641 | Yes |
| 44 | SERINC5 |  |  | 140 | 0.413 | 0.2683 | Yes |
| 45 | RTKN2 |  |  | 141 | 0.413 | 0.2749 | Yes |
| 46 | GTSE1 |  |  | 146 | 0.411 | 0.2766 | Yes |
| 47 | CCNB2 |  |  | 147 | 0.411 | 0.2831 | Yes |
| 48 | ANLN |  |  | 152 | 0.407 | 0.2848 | Yes |
| 49 | MAD2L1 |  |  | 153 | 0.406 | 0.2913 | Yes |
| 50 | LINC01224 |  |  | 154 | 0.406 | 0.2977 | Yes |
| 51 | PBX3 |  |  | 157 | 0.404 | 0.3018 | Yes |
| 52 | EIF4EBP2 |  |  | 158 | 0.403 | 0.3082 | Yes |
| 53 | ARHGAP11A |  |  | 162 | 0.399 | 0.3109 | Yes |
| 54 | KIF2C |  |  | 165 | 0.397 | 0.3149 | Yes |
| 55 | MCM7 |  |  | 171 | 0.394 | 0.3151 | Yes |
| 56 | NDC80 |  |  | 173 | 0.393 | 0.3202 | Yes |
| 57 | CKLF |  |  | 183 | 0.388 | 0.3155 | Yes |
| 58 | HNRNPH3 |  |  | 185 | 0.387 | 0.3204 | Yes |
| 59 | KIF22 |  |  | 197 | 0.377 | 0.3131 | Yes |
| 60 | PARD3 |  |  | 200 | 0.375 | 0.3167 | Yes |
| 61 | CDCA3 |  |  | 203 | 0.372 | 0.3202 | Yes |
| 62 | PTMA |  |  | 206 | 0.371 | 0.3237 | Yes |
| 63 | SMC4 |  |  | 217 | 0.367 | 0.3175 | Yes |
| 64 | RAB13 |  |  | 220 | 0.366 | 0.3209 | Yes |
| 65 | SPC25 |  |  | 226 | 0.363 | 0.3207 | Yes |
| 66 | NCAPH |  |  | 232 | 0.359 | 0.3203 | Yes |
| 67 | ZNF704 |  |  | 235 | 0.357 | 0.3236 | Yes |
| 68 | H2AFZ |  |  | 245 | 0.351 | 0.3183 | Yes |
| 69 | DDIAS |  |  | 253 | 0.348 | 0.3154 | Yes |
| 70 | FEN1 |  |  | 259 | 0.343 | 0.3149 | Yes |
| 71 | TTK |  |  | 262 | 0.340 | 0.3179 | Yes |
| 72 | TUBB |  |  | 264 | 0.340 | 0.3221 | Yes |
| 73 | SLC25A5 |  |  | 266 | 0.340 | 0.3263 | Yes |
| 74 | CENPJ |  |  | 270 | 0.339 | 0.3281 | Yes |
| 75 | SNRPE |  |  | 271 | 0.338 | 0.3335 | Yes |
| 76 | LRR1 |  |  | 273 | 0.337 | 0.3376 | Yes |
| 77 | CLSPN |  |  | 274 | 0.336 | 0.3430 | Yes |
| 78 | DEPDC1B |  |  | 275 | 0.335 | 0.3483 | Yes |
| 79 | ESCO2 |  |  | 282 | 0.333 | 0.3464 | Yes |
| 80 | ORC6 |  |  | 283 | 0.333 | 0.3517 | Yes |
| 81 | UPF2 |  |  | 289 | 0.329 | 0.3509 | Yes |
| 82 | C21orf58 |  |  | 294 | 0.328 | 0.3513 | Yes |
| 83 | MND1 |  |  | 304 | 0.324 | 0.3456 | Yes |
| 84 | RPS6 |  |  | 306 | 0.324 | 0.3495 | Yes |
| 85 | RAD18 |  |  | 307 | 0.324 | 0.3547 | Yes |
| 86 | BRCA2 |  |  | 308 | 0.323 | 0.3598 | Yes |
| 87 | CENPN |  |  | 310 | 0.323 | 0.3638 | Yes |
| 88 | HNRNPDL |  |  | 315 | 0.320 | 0.3641 | Yes |
| 89 | FANCI |  |  | 319 | 0.319 | 0.3655 | Yes |
| 90 | DNA2 |  |  | 321 | 0.318 | 0.3694 | Yes |
| 91 | RFC3 |  |  | 337 | 0.314 | 0.3563 | Yes |
| 92 | CDCA5 |  |  | 341 | 0.312 | 0.3576 | Yes |
| 93 | CLIC1 |  |  | 342 | 0.311 | 0.3626 | Yes |
| 94 | CHD7 |  |  | 343 | 0.311 | 0.3675 | Yes |
| 95 | PRC1 |  |  | 350 | 0.310 | 0.3652 | Yes |
| 96 | POLR3K |  |  | 351 | 0.309 | 0.3702 | Yes |
| 97 | ANP32B |  |  | 357 | 0.307 | 0.3690 | Yes |
| 98 | SLC20A1 |  |  | 361 | 0.306 | 0.3702 | Yes |
| 99 | CCNF |  |  | 366 | 0.305 | 0.3703 | Yes |
| 100 | TTF2 |  |  | 367 | 0.305 | 0.3751 | Yes |
| 101 | CDCA2 |  |  | 369 | 0.304 | 0.3788 | Yes |
| 102 | BRCA1 |  |  | 373 | 0.302 | 0.3800 | Yes |
| 103 | CKAP2 |  |  | 379 | 0.301 | 0.3787 | Yes |
| 104 | RPA3 |  |  | 381 | 0.300 | 0.3823 | Yes |
| 105 | CSE1L |  |  | 382 | 0.300 | 0.3871 | Yes |
| 106 | RRM2 |  |  | 383 | 0.300 | 0.3919 | Yes |
| 107 | XRCC2 |  |  | 384 | 0.300 | 0.3966 | Yes |
| 108 | CENPW |  |  | 386 | 0.299 | 0.4002 | Yes |
| 109 | TEX30 |  |  | 388 | 0.298 | 0.4038 | Yes |
| 110 | CDC6 |  |  | 391 | 0.297 | 0.4061 | Yes |
| 111 | HMGA1 |  |  | 397 | 0.295 | 0.4047 | Yes |
| 112 | RACGAP1 |  |  | 399 | 0.293 | 0.4082 | Yes |
| 113 | PA2G4 |  |  | 402 | 0.293 | 0.4105 | Yes |
| 114 | HMGB1 |  |  | 403 | 0.292 | 0.4151 | Yes |
| 115 | HMGB2 |  |  | 424 | 0.288 | 0.3955 | Yes |
| 116 | PID1 |  |  | 429 | 0.287 | 0.3953 | Yes |
| 117 | NUSAP1 |  |  | 430 | 0.286 | 0.3998 | Yes |
| 118 | NUF2 |  |  | 434 | 0.285 | 0.4007 | Yes |
| 119 | NCAPD2 |  |  | 440 | 0.283 | 0.3992 | Yes |
| 120 | LMNB1 |  |  | 442 | 0.282 | 0.4025 | Yes |
| 121 | CDC25C |  |  | 443 | 0.282 | 0.4070 | Yes |
| 122 | CDC45 |  |  | 446 | 0.282 | 0.4091 | Yes |
| 123 | ANP32E |  |  | 453 | 0.280 | 0.4063 | Yes |
| 124 | BCHE |  |  | 456 | 0.280 | 0.4083 | Yes |
| 125 | GINS1 |  |  | 462 | 0.279 | 0.4067 | Yes |
| 126 | MNS1 |  |  | 464 | 0.278 | 0.4100 | Yes |
| 127 | ATAD2 |  |  | 470 | 0.275 | 0.4083 | Yes |
| 128 | EZH2 |  |  | 471 | 0.275 | 0.4127 | Yes |
| 129 | SKA1 |  |  | 475 | 0.274 | 0.4134 | Yes |
| 130 | SHCBP1 |  |  | 477 | 0.274 | 0.4166 | Yes |
| 131 | NHLRC2 |  |  | 488 | 0.272 | 0.4088 | No |
| 132 | BLM |  |  | 490 | 0.272 | 0.4120 | No |
| 133 | KIF4A |  |  | 504 | 0.269 | 0.4005 | No |
| 134 | HAUS8 |  |  | 518 | 0.267 | 0.3891 | No |
| 135 | ZNF90 |  |  | 520 | 0.266 | 0.3921 | No |
| 136 | RCC1 |  |  | 528 | 0.265 | 0.3879 | No |
| 137 | SKA3 |  |  | 531 | 0.264 | 0.3897 | No |
| 138 | ALYREF |  |  | 535 | 0.264 | 0.3903 | No |
| 139 | RANBP1 |  |  | 536 | 0.263 | 0.3945 | No |
| 140 | KIF15 |  |  | 538 | 0.263 | 0.3974 | No |
| 141 | SRSF2 |  |  | 542 | 0.262 | 0.3980 | No |
| 142 | STIL |  |  | 545 | 0.261 | 0.3997 | No |
| 143 | CENPO |  |  | 549 | 0.260 | 0.4003 | No |
| 144 | FBXO5 |  |  | 557 | 0.258 | 0.3959 | No |
| 145 | DDX39A |  |  | 562 | 0.258 | 0.3952 | No |
| 146 | TMPO |  |  | 584 | 0.253 | 0.3738 | No |
| 147 | ABAT |  |  | 617 | -0.259 | 0.3393 | No |
| 148 | FAM111A |  |  | 618 | -0.259 | 0.3434 | No |
| 149 | MAGI1 |  |  | 642 | -0.266 | 0.3198 | No |
| 150 | C6orf62 |  |  | 655 | -0.272 | 0.3097 | No |
| 151 | RAB3IP |  |  | 667 | -0.280 | 0.3008 | No |
| 152 | CKB |  |  | 676 | -0.287 | 0.2957 | No |
| 153 | AKAP12 |  |  | 690 | -0.296 | 0.2847 | No |
| 154 | ZEB2 |  |  | 722 | -0.319 | 0.2523 | No |
| 155 | GRIK3 |  |  | 747 | -0.341 | 0.2288 | No |
| 156 | DLX1 |  |  | 770 | -0.361 | 0.2079 | No |
| 157 | MSI2 |  |  | 771 | -0.361 | 0.2137 | No |
| 158 | TMSB4X |  |  | 850 | -0.443 | 0.1264 | No |
| 159 | TFDP2 |  |  | 858 | -0.448 | 0.1251 | No |
| 160 | RND3 |  |  | 959 | -0.864 | 0.0180 | No |
| 161 | CDK6 |  |  | 966 | -0.920 | 0.0254 | No |
Table: GSEA details [plain text format]

  

Fig 2: RICHARDS\_NOWAKOWSKI\_SCIENCE\_2017\_MGE\_DIV\_UPREG: Random ES distribution      
 Gene set null distribution of ES for **RICHARDS\_NOWAKOWSKI\_SCIENCE\_2017\_MGE\_DIV\_UPREG**
